# Untangling the Conformational Polymorphism of Disordered Proteins Associated With Neurodegeneration at the Single-Molecule Level

**DOI:** 10.3389/fnmol.2019.00309

**Published:** 2020-01-10

**Authors:** Melissa Birol, Ana M. Melo

**Affiliations:** ^1^Department of Chemistry, University of Pennsylvania, Philadelphia, PA, United States; ^2^Centro de Química-Física Molecular- IN and iBB-Institute for Bioengineering and Biosciences, Instituto Superior Técnico, Universidade de Lisboa, Lisbon, Portugal

**Keywords:** intrinsically disordered proteins, neurodegenerative diseases, single-molecule FRET, fluorescence correlation spectroscopy, huntingtin exon 1, tau, α-synuclein

## Abstract

A large fraction of the human genome encodes intrinsically disordered proteins/regions (IDPs/IDRs) that are involved in diverse cellular functions/regulation and dysfunctions. Moreover, several neurodegenerative disorders are associated with the pathological self-assembly of neuronal IDPs, including tau [Alzheimer’s disease (AD)], α-synuclein [Parkinson’s disease (PD)], and huntingtin exon 1 [Huntington’s disease (HD)]. Therefore, there is an urgent and emerging clinical interest in understanding the physical and structural features of their functional and disease states. However, their biophysical characterization is inherently challenging by traditional ensemble techniques. First, unlike globular proteins, IDPs lack stable secondary/tertiary structures under physiological conditions and may interact with multiple and distinct biological partners, subsequently folding differentially, thus contributing to the conformational polymorphism. Second, amyloidogenic IDPs display a high aggregation propensity, undergoing complex heterogeneous self-assembly mechanisms. In this review article, we discuss the advantages of employing cutting-edge single-molecule fluorescence (SMF) techniques to characterize the conformational ensemble of three selected neuronal IDPs (huntingtin exon 1, tau, and α-synuclein). Specifically, we survey the versatility of these powerful approaches to describe their monomeric conformational ensemble under functional and aggregation-prone conditions, and binding to biological partners. Together, the information gained from these studies provides unique insights into the role of *gain* or *loss of function* of these disordered proteins in neurodegeneration, which may assist the development of new therapeutic molecules to prevent and treat these devastating human disorders.

## Introduction

The classical protein “structure–function” paradigm establishes that proteins fold into a unique ordered 3D structure determined by their amino acid sequence before acquiring a specific biological function (reviewed in Fersht, 2008). However, studies over the last two decades have identified functional proteins lacking a stable secondary and/or tertiary structure, and instead adopting a dynamic ensemble of multiple conformational states (Kriwacki et al., 1996; Wright and Dyson, 1999; Mittag et al., 2010; Babu et al., 2012; Tompa, 2012; van der Lee et al., 2014). These intrinsically disordered proteins and regions (IDPs and IDRs, respectively) are widespread in the human proteome and play critical roles in diverse biological processes, including in transcription and translation, cell cycle, signaling, and transport (Iakoucheva et al., 2002; Wright and Dyson, 2015; Babu, 2016; Tsafou et al., 2018). Their functional diversity is sustained by unique features: (i) quick response to variations in cellular environment; (ii) interaction with multiple binding partners (with high specificity but low affinity) that provides binding promiscuity; and (iii) tight regulation by posttranslational modifications (PTMs) (Babu et al., 2012; Uversky, 2015; Babu, 2016). The misbehavior and misfolding of these naturally flexible proteins or regions can ultimately lead to their dysfunction. Therefore, IDPs/IDRs have been implicated in several devastating human diseases (such as in neurodegeneration and cancer), supporting the emerging “disorder in disorders” (or D^2^) concept (Uversky et al., 2008).

Many severe neurodegenerative disorders are associated with the pathological self-assembly and extra- or intracellular deposition of neuronal IDPs or proteins containing IDRs. These include amyloid-β (Aβ) peptides in Alzheimer’s disease (AD); tau in multiple tauopathies (including AD); α-synuclein (αS) in Parkinson’s disease (PD); huntingtin (HTT) in Huntington’s disease (HD); and TAR DNA-binding protein-43 (TDP-43) and fused in sarcoma (FUS) protein in amyotrophic lateral sclerosis and frontotemporal lobar degeneration (FTLD; reviewed in Uversky, 2014, 2015). These neurodegeneration-promoting proteins are fully or locally disordered in their monomeric-unbound state, but surprisingly they display a high tendency to form ordered insoluble aggregates (Uversky, 2014). In addition, accumulated evidence supports that aggregation-prone IDPs/IDRs can cause neurodegeneration through the failure to adopt a functional state (loss of their native functions) and/or gain of abnormal toxic interactions or protein accumulation resulting in toxic oligomers/aggregates (toxic gain of function; Trojanowski and Lee, 2005; Winklhofer et al., 2008). Effective therapies should be designed to restore their biological functions and/or avoid their aggregation at early stages. Therefore, there is an urgent medical interest in understanding these neuronal IDPs/IDRs in normal and disease conditions by: (i) characterizing their functional diversity and structural rearrangements upon interaction with distinct binding partners; and (ii) determining the conformational ensemble of their monomers under conditions that favor aggregation, which are ideal clinical targets. Together, these approaches will provide insights into the key physical and structural features of these aggregation-prone IDPs/IDRs that trigger gain or loss of function, and ultimately cause neuronal cell death.

**Figure 1:**
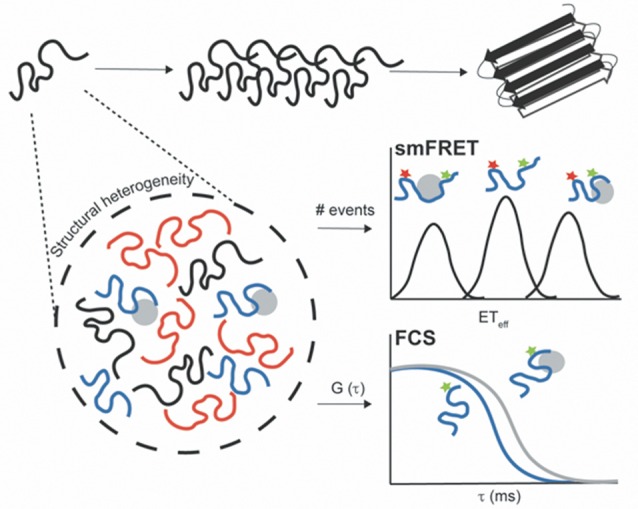
Untangling the conformational heterogeneity and molecular interactions of monomeric intrinsically disordered proteins (IDPs) through single-molecule fluorescence (SMF) approaches. Single-molecule Förster resonance energy transfer (smFRET) histogram discriminating between distinct conformational ensembles of double-label protein (donor–acceptor): monomeric free protein in solution and bound states with extended (low ET_eff_) or compacted conformations (high ET_eff_). Fluorescence correlation spectroscopy (FCS) autocorrelation curve reporting on changes in the diffusion time of single-label protein free in solution (blue curve) and upon interaction with aggregation inducers or binding partners (gray curve). The binding results into a shift of the autocorrelation curve to the right (longer translational diffusion times).

Remarkably, single-molecule fluorescence (SMF) methods have enhanced our understanding of IDPs, including amyloid-forming proteins, during the past two decades (reviewed in Brucale et al., 2014; Schuler et al., 2016). Numerous SMF methodologies have been developed for probing transient oligomeric species and to determine their stoichiometry. These include two-color coincidence detection (TCCD; Cremades et al., 2012), single-molecule Förster resonance energy transfer (smFRET; Shammas et al., 2015), total internal reflection fluorescence microscopy (TIRF)-based approaches (Lv et al., 2015), or single-molecule photobleaching (Zijlstra et al., 2012). In addition, SMF techniques have been successfully used to characterize the complex conformational distribution and plasticity of monomeric IDPs, and molecular interactions with biological partners or aggregation inducers (Banerjee and Deniz, 2014; Lee et al., 2015). While the application of SMF approaches to uncover oligomeric states has been largely debated (Kundel et al., 2018), in this review article, we focus on the use of SMF methods to study the conformational ensemble of monomeric neurodegeneration-promoting IDPs under functional and aggregation-prone conditions. Notably, the low protein concentrations required for these techniques (in the pM or nM range) inhibit the rapid protein self-assembly. In addition, recording behaviors of individual molecules enables the description of dynamic/heterogeneous systems and the detection of coexisting subpopulations, which are not accessible in traditional ensemble and time-averaging methodologies (Figure 1; Joo et al., 2008; Schuler and Eaton, 2008; Schuler et al., 2016). From this class of SMF methods, fluorescence correlation spectroscopy (FCS) and smFRET have emerged as powerful and versatile tools.

FCS measures fluorescence-intensity fluctuations arising from the diffusion of a few fluorescent molecules through a small confocal observation volume (~1 fL). These fluorescence fluctuations are then analyzed using the autocorrelation function that quantifies the self-similarity of the signal over several delay times. Commonly, it can provide information in local concentrations, molecular mobility, and/or photophysical properties (Hess et al., 2002). Moreover, FCS allows to determine the overall chain dimensions of proteins (for homogeneous populations) and molecular interactions using the translational diffusion time at a slow time scale and conformational dynamics at fast time scales (Chattopadhyay et al., 2005; Sherman et al., 2008; Melo et al., 2011). In particular, nanosecond FCS in conjugation with smFRET and polymer physics can be used to quantify the reconfiguration time of unfolded polypeptide chains (Soranno et al., 2012). Therefore, FCS has been widely applied to evaluate the hydrodynamic size of monomeric IDPs, internal conformational dynamics, and molecular interactions (Figure 1; Crick et al., 2006; Rhoades et al., 2006; Middleton and Rhoades, 2010; Li et al., 2015; Li and Rhoades, 2017).

In addition, smFRET relies on the non-radiative energy transfer from a donor fluorophore to an acceptor fluorophore through a dipole–dipole coupling mechanism. The efficiency of transfer (ET_eff_) exhibits an inverse sixth power dependence on the interdye distance, allowing to determine distances on the nanometer scale (~2–10 nm, as a “spectroscopic ruler”; Forster, 1949). Therefore, intramolecular smFRET (both donor and acceptor fluorophores located on same molecule) can evaluate conformational rearrangements and dynamics of proteins (Chen and Rhoades, 2008; Banerjee and Deniz, 2014; Schuler et al., 2016). Several strategies have been applied for site-specific double-labeling IDPs, including the use of cysteine residues and genetically encoded unnatural amino acids (Lemke, 2011). Briefly, in diffusion-based smFRET, fluorescence intensities are recorded for each donor–acceptor labeled protein while it diffuses across a small confocal volume, and subsequently ET_eff_ values are calculated for each photon burst and plotted as a histogram (Brucale et al., 2014; Schuler et al., 2016). Due to the high structural heterogeneity of IDPs, the Förster equation cannot provide a precise conversion of mean ET_eff_ values into distances (O’Brien et al., 2009), since there is a wide distribution of donor–acceptor distances (without a single fixed distance). Commonly, several polymer models have been employed to describe the conformational ensemble of IDPs and denatured proteins. These include the Gaussian chain, worm-like chain, or a weighted Flory-Fisk distribution (discussed in detail in Schuler et al., 2016). Remarkably, intramolecular smFRET can report on conformational changes and dynamics of neurodegeneration-promoting IDPs in solution and upon binding to functional partners or aggregation promoters (Figure 1; Ferreon et al., 2009; Trexler and Rhoades, 2009, 2010; Elbaum-Garfinkle and Rhoades, 2012; Melo et al., 2016, 2017).

In this short review, we focus on the IDPs—huntingtin exon 1 (HTTex1), tau, and αS—and discuss a series of key SFM studies to characterize their heterogeneous/dynamic monomeric conformational ensemble and also interactions relevant for their function or disease.

## HTTex1 in HD

HTT is a large multidomain protein (with over 3,000 amino acids and 348 kDa) that is involved in several complex cellular processes, such as in trafficking of vesicles and organelles, transcription regulation, and generally in cellular homeostasis (reviewed in Schulte and Littleton, 2011; Saudou and Humbert, 2016). HTT is of major clinical relevance because the abnormal expansion of the CAG repeat within the first exon of its gene (IT15) is the pathological hallmark of HD (MacDonald et al., 1993). Above a critical threshold of about 37 CAG repeats, it leads to the expression of a mutant HTT protein with an expanded polyglutamine (polyQ) domain, which ultimately forms amyloid-like fibrils and intracellular inclusion bodies (Bates et al., 2014). The aberrant splicing and proteolytic cleavage of mutant proteins result in highly toxic HTT fragments spanning exon 1 (HTTex1; Wellington et al., 2002; Sathasivam et al., 2013), which are sufficient to replicate much of HD’s pathology/progression (Mangiarini et al., 1996). The exact molecular mechanism whereby HTTex1 contributes to neurodegeneration remains elusive, but growing evidence supports that the molecular sources of neurotoxicity inherent to the polyQ expansion are toxic conformations of the monomer and/or oligomers (Nagai et al., 2007; Takahashi et al., 2008). Therefore, considerable effort has been devoted to characterize the conformational features of monomeric polyQ peptides and HTTex1 in solution (reviewed in Wetzel, 2012; Adegbuyiro et al., 2017).

HTTex1 consists of a polyQ domain flanked by an N-terminal 17 amino acid segment (N17) and a C-terminal proline-rich region (PRR; Wetzel, 2012; Adegbuyiro et al., 2017). Since the age of onset, risk of disease and severity in HD are strongly correlated with the polyQ length (Bates et al., 2014), the early structural studies solely focused on simple synthetic polyQ peptides (with extra lysine residues to improve their solubility). Several circular dichroism (CD) and nuclear magnetic resonance (NMR) studies have shown that simple polyQ sequences regardless of their repeat length are predominantly disordered in solution (Altschuler et al., 1997; Chen et al., 2001; Klein et al., 2007). In a pioneering FCS study, Crick et al. (2006) identified the hydrodynamic radius of monomeric polyQ peptides to obtain insights into their global dimensions and also shapes. The authors measured the average translational diffusion time (proportional to the hydrodynamic radius) of (Gly)-(Gln)_N_-Cys-Lys_2_ peptides in solution and assessed how it scales with the chain length. Notably, FCS revealed that: (i) aqueous solution is a “poor solvent” (low scaling exponent with *v* = 0.32 ± 0.02), suggesting that monomeric polyQ sequences adopt a heterogeneous ensemble of collapsed conformations; and (ii) the absence of sharp structural transitions across the critical polyQ length (no drastic changes in the diffusion time with increasing chain lengths).

A detailed understanding of the structural basis of monomeric mutant HTTex1 is crucial for developing a mechanistic model for HTTex1 toxicity. Current hypotheses describing the toxic structural threshold are largely sustained by indirect evidences. In particular, numerous studies over the last decade support that both flanking regions (PRR and N17) strongly modulate the aggregation of HTTex1 in solution (Thakur et al., 2009; Crick et al., 2013; Shen et al., 2016) and in the presence of biological membranes (Burke et al., 2013). Specifically, in solution, N17 enhances aggregation in a distinct mechanism to that of synthetic polyQ peptides (Thakur et al., 2009), while PRR displays an opposite effect, favoring aggregation-resistant conformations (Bhattacharyya et al., 2006). Together, it supports that the cross-talk between both flanking regions and/or a sharp conformational transition above the pathological polyQ threshold control the HTTex1 cytotoxicity. Nevertheless, there remains controversy due to the absence of single-atom resolution structures of HTTex1 (ones lacking solubilizing tags or stabilizing amino acids). This is due to the high aggregation propensity of HTTex1, the disordered features of the polyQ stretches, and the inherent challenge of recombinant expression and purification of HTTex1. While a recent high-resolution cryo-electron microscopy structure for the full-length HTT protein was reported in a complex with HTT-associated protein 40 (HAP40), the exon 1 region was not solved due to its disordered nature (Guo et al., 2018). Notably, the first single-molecule structural characterization of monomeric HTTex1 in solution was recently provided by a collaborative study from the Lemke, Pappu, and Lashuel groups (Warner et al., 2017). In this elegant study, smFRET was used to determine intramolecular distances within monomeric HTTex1 at pM concentrations, where its self-assembly and phase separation are prevented. Briefly, both an intein-fusion strategy and a semi-synthetic approach were employed to create five polyQ lengths HTTex1 variants (15Q, 23Q, 37Q, 43Q, and 49Q). For each variant, multiple double-labeled smFRET constructs were designed by site-specific labeling at a fixed position in N17 (A2C with Alexa 488 maleimide dye) and variable in PRR (A60C, P70C, P80C, or P90C with Alexa 594 maleimide dye). Remarkably, using smFRET in combination with atomistic simulations, Warner and co-workers proposed that both wild-type (WT) and mutant HTTex1 adopt a “tadpole-like” topology, in which N17 adsorbs on the polyQ tract, making a “globular head,” and the PRR domain forms an extended/semi-flexible chain. Therefore, contrary to previous indirect evidences, smFRET data argue against sharp structural transitions in HTTex1 at pathological polyQ lengths in solution. The authors suggested that the increase of the polyQ surface area with its length promotes: (i) toxic “heterotypic interactions” by increasing the binding sites; and (ii) “homotypic interactions” that ultimately trigger HTTex1 aggregation.

In light of this recent smFRET work, future studies should firstly evaluate whether the toxic polyQ expansion controls the HTTex1 interactome. In particular, FCS or fluorescence cross-correlation spectroscopy (FCCS) will allow to quantify the interaction of WT (as control) and mutant HTTex1 with: (i) biological partners or emerging interacting proteins; (ii) biological membranes with variable lipid composition; and (iii) molecular chaperones. Simultaneously, the characterization of the conformational ensemble of HTTex1 under functional and aggregation-prone conditions through smFRET will provide insights into toxicity relevant conformations.

## Tau in Tauopathies

Tau is a microtubule-associated protein (MAP) found predominantly in the axons of neurons (Litman et al., 1993; Hirokawa et al., 1996) that plays a critical role in microtubule (MT) assembly/stabilization (Weingarten et al., 1975; Drubin and Kirschner, 1986; Gustke et al., 1994; Trinczek et al., 1995; Goode et al., 2000) and axonal transport (Ebneth et al., 1998; Terwel et al., 2002). Its pathological aggregation and deposition are associated with numerous devastating neurodegenerative disorders termed tauopathies, including AD, FTLD, chronic traumatic encephalopathy, and Pick’s disease (reviewed in Brunden et al., 2009). Accumulating evidence supports that the disruption of its native function as a MAP can also contribute to these tauopathies (Ballatore et al., 2007; Winklhofer et al., 2008). For instance, the abnormal hyperphosphorylation of tau can promote its self-assembly into toxic oligomers and paired helical filaments (PHFs), as well as reduces tau–MT interaction, resulting in MT destabilization and cell death (reviewed in Johnson and Stoothoff, 2004).

Tau consists of three major functional regions: (1) MT binding region (MTBR) composed of imperfect repeats that directly interacts with MTs/tubulin (Butner and Kirschner, 1991) and forms the core of PHFs (Crowther et al., 1989); (2) a proline-rich domain (PRD) that increases MT-binding/assembly (Gustke et al., 1994); and finally (3) an N-terminal projection domain that controls MT spacing (Chen et al., 1992) and might bind to neuronal plasma membrane (Brandt et al., 1995). In adult human brains, the alternative splicing of a single MAPT gene results in six different isoforms (ranging from 352 to 441 amino acids) that contain up to two N-terminal inserts (0N, 1N, and 2N) and three or four imperfect repeats (3R or 4R) within MTBR.

Tau is a large disordered protein in its monomeric unbound state (Cleveland et al., 1977). The full-length protein is highly demanding for NMR (Mukrasch et al., 2009), and so far, most NMR studies have used MTBR fragments. In a seminal ensemble FRET study, Mandelkow and co-workers identified that tau forms a highly compact structure in solution described by a “paperclip” conformation (Jeganathan et al., 2006). Specifically, this early work revealed that the C-terminus is in close proximity to the N-terminus and the MTBR, but without a measured FRET distance (so higher than 10 nm) between the N-terminus and the MTBR. Recently, the Rhoades Lab used SMF methods to characterize the aggregation-prone structures of tau [in the presence of heparin (Elbaum-Garfinkle and Rhoades, 2012) and polyphosphates (polyP; Wickramasinghe et al., 2019)] and the functional conformations [with soluble tubulin heterodimers (Elbaum-Garfinkle et al., 2014; Li et al., 2015; Melo et al., 2016)]. Below, we summarize the studies reporting on the conformational transitions undergone by tau relevant to its functional and aggregation-prone states.

Initial smFRET work by Elbaum-Garfinkle and Rhoades redefined the “paperclip” model from ensemble measurements for tau monomer in solution and also characterized its conformational ensemble in the presence of heparin (Elbaum-Garfinkle and Rhoades, 2012). This comprehensive work investigated 12 double-labeled constructs of tau that mapped multiple overlapping regions within the 2N4R isoform (longest tau isoform), and their respective ET_eff_ were reported for each construct in the absence and presence of heparin. In solution, the overall dimensions of tau diverge from a theoretical random coil protein in a “good solvent.” This is further sustained by intramolecular contacts between the N- and C-termini and also each terminus and the MTBR, which was ascribed to electrostatic effects. Together, smFRET data supported that tau adopts more an “S-shaped” than a “paperclip” topology (Jeganathan et al., 2006; from ensemble FRET) in solution, since the MTBR is in relatively close contact with both termini. The discrepancy is explained, in part, by the use of: (i) a donor–acceptor pair with a small Förster radius; and (ii) the Förster equation to directly convert ET_eff_ into distances, in ensemble measurements. Notably, the same work also revealed that tau undergoes a two-state conformational transition upon binding to heparin underlined by the pronounced MTBR compaction and the loss of the long-range interactions between the two termini. Moreover, different domains of tau exhibit distinct physical and structural features, and consequently they respond differentially upon heparin binding. In a recent study, Wickramasinghe and co-workers investigated the interaction of tau with the physiologically aggregation inducer, polyP, using FCS and smFRET (Wickramasinghe et al., 2019). Specifically, following a similar approach as described for heparin, smFRET data reported that polyP promotes a local compaction of the MTBR and PRD, with a concomitant decrease in the long-range contacts between both termini. The binding of tau to polyP was further characterized by FCS, revealing that both PRD and MTBR interact with polyP. The conformational changes and aggregation effects depicted were found to strongly correlate with the polyP chain length. Moreover, longer polyP chains were shown to promote intermolecular interactions in tau monomers (working as “intermolecular scaffold”), thus inducing its pathological aggregation.

Most research on tau function has been focused so far on its role in MT dynamic instability and its interaction with stabilized MTs. In addition, ensemble MT polymerization assays (based on scatter measurements) do not provide a detailed description of the first step of MT assembly. Therefore, the molecular mechanism by which tau promotes the polymerization of tubulin into MTs remains poorly understood. The Rhoades Lab has applied SMF methods to describe the largely overlooked interaction of tau with soluble tubulin heterodimers (the first step of MT assembly mechanism). In a pioneering work, Elbaum-Garfinkle et al. (2014) identified for the first time by FCS that tau binds to soluble tubulin heterodimers (under non MT assembly conditions, with a low concentration of tau and in a buffer lacking GTP), and disease mutations also enhance this interaction. In two subsequent FCS studies, Li et al. (2015) and Li and Rhoades (2017) showed that: (i) tau binds to multiple tubulin heterodimers (Li et al., 2015); and (ii) the C-terminal pseudo-repeat region of tau (adjacent to MTBR) increases the heterogeneity of tau–tubulin complex with further independent binding sites at R2 and R3, in which the size and heterogeneity are strongly linked to tau function (MT polymerization; Li and Rhoades, 2017). The topological features of tau in this heterogeneous/dynamic complex were further investigated by intramolecular smFRET (Melo et al., 2016). These measurements were performed under 100% tubulin binding and a multiprobe approach was again employed for 2N4R and 2N3R tau isoforms. Remarkably, large shifts toward lower mean ET_eff_ were observed for constructs probing the solution long-range interactions (for both termini and each terminus and the MTBR). This work revealed that tau adopts an overall open structure in this complex, exposing binding sites within the MTBR. Similarly, this expansion is observed upon binding to the MT surface (Sillen et al., 2007) and heparin (Elbaum-Garfinkle and Rhoades, 2012). Surprisingly, smFRET data also showed that the MTBR conserves its global dimensions, while its individual repeats experience local extensions to provide binding to multiple tubulin heterodimers. The extent of conformational changes within the MTBR was larger for two repeat-spanning constructs for both isoforms, also including R3. Contrary to NMR data for tau bound to a single-tubulin dimer (Gigant et al., 2014), no evidence for the U-turn topology adopted by the MTBR was found. Finally, these findings supported that tau forms a “fuzzy complex” with soluble tubulin, in which it retains its flexibility and conformational plasticity as an IDP. However, it remains to be elucidated whether tau can bind simultaneously tubulin and MTs.

In summary, smFRET revealed that the MTBR responds differentially upon interaction with soluble tubulin or heparin/polyP that accounts for distinct conformational ensembles of tau in its tubulin-bound state and aggregation-prone structure, respectively. In addition, it provides a framework to explore the role of PTMs (as hyperphosphorylation) in the conformational ensemble of monomeric tau under functional (“fuzzy complex” with soluble tubulin) and disease conditions. Finally, as recent studies support that tau can undergo liquid–liquid phase separation (LLPS; Hernández-Vega et al., 2017; Zhang et al., 2017; Wegmann et al., 2018), smFRET provides a versatile tool to probe early conformational changes that trigger phase separation (in the presence of molecular crowding and RNA), and to explore the dynamics within the droplet.

## αS in PD

αS is a small protein (140 amino acids) abundantly expressed in presynaptic terminals of neurons in the human brain (Maroteaux et al., 1988; Jakes et al., 1994). This protein is intrinsically flexible in solution and, similar to other IDPs, acquires structure upon binding to biological binding partners as lipid membranes (Weinreb et al., 1996; Davidson et al., 1998; Eliezer et al., 2001). While αS has been associated with several biological activities, including in regulation of synaptic vesicles pools (Murphy et al., 2000; Cabin et al., 2002), neurotransmitter release (Nemani et al., 2010), SNARE complex assembly (Burré et al., 2010), and vesicle trafficking (Cooper et al., 2006; Snead and Eliezer, 2019), the precise function of this protein remains enigmatic and controversial. αS is the major component of intracellular amyloid deposits known as Lewy bodies and has thus been implicated in the development and pathogenesis of neurodegenerative disorders, such as PD (Polymeropoulos et al., 1997; Spillantini et al., 1998; Goedert, 2001). In particular, biological membranes appear to play a key role in αS function and dysfunction (reviewed in Snead and Eliezer, 2014).

αS contains three main regions: (1) N-terminal region that mediates binding to lipid membranes; (2) a central hydrophobic non-amyloid β-component (NAC) region responsible for its self-assembly; and (3) a highly negatively charged C-terminus. Several ensemble biophysical methods, including NMR (Eliezer et al., 2001; Bussell and Eliezer, 2003; Chandra et al., 2003; Dedmon et al., 2005), electron paramagnetic resonance (EPR) spectroscopy (Jao et al., 2004; Drescher et al., 2008), and CD (Chandra et al., 2003; Ferreon and Deniz, 2007), have provided valuable insights into the conformational transitions in αS upon interaction with sodium dodecyl sulfate (SDS) micelles and/or lipid vesicles. In addition, a recent in-cell NMR study revealed that the disordered nature of monomeric αS is highly conserved in the cytoplasm of mammalian cells (Theillet et al., 2016).

A seminal FCS work from the Webb and Eliezer groups quantified the binding of αS to large unilamellar vesicles (LUVs) prepared with variable anionic lipid content, showing that electrostatic effects strongly enhance the αS-lipid interaction (Rhoades et al., 2006). In a subsequent FCS study from the Rhoades lab, this interaction was further explored as a function of different lipid compositions (anionic and saturated lipids), membrane curvature, and PD-associated mutations (Middleton and Rhoades, 2010). This work revealed a preferential binding of αS to gel-phase liposomes when compared to fluid-phase vesicles. Moreover, it reported on drastic effects of membrane curvature, underlining a stronger affinity of αS for small unilamellar vesicles (SUVs) over LUVs. Finally, PD-associated mutations presented only minor changes in the molar membrane-partition coefficients compared to the WT protein.

To better understand the disorder-to-order transitions in αS, several studies have employed SMF methods to identify and resolve multiple coexisting populations and its structural heterogeneity. Pioneering smFRET experiments from the Deniz, Rhoades, and Subramaniam labs provided structural insights into the conformational switching between the broken and extended α-helical structures adopted by αS upon binding to SDS micelles and lipid vesicles (Ferreon et al., 2009; Trexler and Rhoades, 2009; Veldhuis et al., 2009). These works were able to distinguish between conflicting reports from ensemble measurements debating the configuration of micelle or lipid bound αS (Chandra et al., 2003; Borbat et al., 2006). smFRET identified a broken α-helical conformation adopted by αS upon binding to SDS above the critical micelle concentration. Further, the Deniz and Rhoades labs identified that the binding surface curvature strongly modulates the helical topology of αS. Their works revealed that αS adopts an extended helical structure upon binding to low-curvature SDS or lipid surfaces, while it assumes a bent-helix conformation on highly curved SDS micelles. Further work from the Deniz group explored the effect of PD-associated mutations on αS folding by both CD and smFRET measurements (Ferreon et al., 2010). It revealed that A53T, E46K, and C-terminal truncation (residues 1–107) variants display a similar multistate folding behavior to WT protein. However, the A30P mutation (located on the membrane binding region) was found to not adopt an extended conformation at SDS concentrations near or below the critical micelle concentration. A more recent study from the same lab investigated the “two-dimensional (2D) crowding” effect on the structural transitions of αS at membrane surface (Banerjee et al., 2016). Under high “2D crowding” conditions promoted by the simultaneous membrane binding of αS and Hsp27 (a lipid-interacting chaperone), αS was found to adopt an alternative (“hidden”) conformation, which is not highly populated at chaperone-free conditions.

smFRET work from Trexler and Rhoades also probed the aggregation-prone structures of αS under different aggregation-promoting conditions, such as low pH and in the presence of aggregation inducers (spermine and heparin; Trexler and Rhoades, 2010). Remarkably, this work revealed that the low pH and aggregation inducers promote distinct effects on the αS structure. Briefly, the C-terminus of αS was found to structurally collapse at low pH, while minor effects were reported on the N-terminus and the central region of the protein. However, this local compaction of C-terminal region had no significant effect on the overall dimensions of αS. Meanwhile, αS binding to both heparin or spermine showed a lack of large-scale structural transitions. In addition, recent work from the Deniz lab used a combination of SMF and ensemble methods to investigate the effect of osmolytes in αS folding (Moosa et al., 2015). Contrary to the SDS folding pathway, this study supported that αS follows a two-state transition in the presence of osmolytes, consisting of rapid interconverting conformations of unfolded and force-folded states. The effects obtained for both osmolytes and “2D crowding” support that complex cellular contexts need to be explored in order to describe the physiological folding landscape of αS. On that note, recent work from the Rhoades lab revealed that cell-surface-exposed glycans are potential cellular interactors of αS (Birol et al., 2019). This work employed FCS to quantify the interaction of monomer αS with glycans, and identified these cell exposed glycans as key modulators of αS internalization in cells.

Together, these works have identified and described the conformational landscape of αS under diverse conditions (such as membrane-bound state, PD-associated mutations, and the presence of osmolytes and “2D crowding”) and provide an outline in the future to also evaluate the impact of PTMs. In addition, future *in vivo* smFRET measurements could complement in-cell MNR data (although performed at different concentration range). In particular, it will allow to determine the physical/structural features of αS under physiological and disease situations and to evaluate *in vivo* conditions promoting the disorder-to-order transition.

## Conclusion Remarks

SMF methods have been recognized as powerful and versatile approaches to investigate the heterogeneous and dynamic nature of neurodegeneration-associated IDPs, including HTTex1, tau, and αS as discussed in this review article. These cutting-edge techniques have provided valuable insights into: (i) their monomeric states; (ii) the aggregation-prone structures of tau and αS; (iii) the disorder-to-order transition of αS upon membrane binding; and finally (iv) the formation of a “fuzzy complex” by tau bound to soluble tubulin. However, the structural interpretation of smFRET data is highly challenging for IDPs due to their heterogeneous and dynamic nature. For IDPs systems, Förster equation does not provide a direct conversation of ET_eff_ in distances, and polymer physics models have been successfully applied to describe the broad distribution of donor–acceptor distances. Moreover, smFRET requires site-specific double-labeling proteins with small organic dyes through natural/mutated cysteines or genetically encoded unnatural amino acids. Meanwhile, molecular dynamics (MD) simulation provides a valuable tool to rationalize smFRET data, including to describe the dynamic movement of the dye molecule and to consider its linker, and together to provide insights into IDP conformational dynamics.

Most SMF research performed for the discussed proteins has been restricted to *in vitro* studies. As such, native functional interactions and the associated conformational changes in the cellular context are underinvestigated. Therefore, it is crucial to move SMF methods towards *in vivo* conditions. For smFRET, it requires developing new strategies for *in vivo* labeling proteins, as fluorescent proteins are not suitable. Recent advances by Schuler and colleagues used microinjection to deliver IDPs (recombinant protein labeled *in vitro*) in live mammalian cells and employed a range of SMF techniques (including smFRET) to describe their structural dynamics (König et al., 2015), providing a new avenue to study this challenging class of proteins. We anticipate that adapting a similar approach to the systems discussed in this review article and future advances in SMF tools and application will allow to characterize the conformational ensemble of these neuronal IDPs *in vivo*, and simultaneously reveal and delineate toxic structural transitions associated to their loss of function or aggregation.

## Author Contributions

AM conceptualized the manuscript. AM and MB conducted the literature review, drafted the manuscript and revised the manuscript.

## Conflict of Interest

The authors declare that the research was conducted in the absence of any commercial or financial relationships that could be construed as a potential conflict of interest.

## References

[B1] Adegbuyiro A. Sedighi F. Pilkington A. W. Groover S. Legleiter J. (2017). Proteins containing expanded polyglutamine tracts and neurodegenerative disease. Biochemistry 56, 1199–1217. 10.1021/acs.biochem.6b00936 28170216 PMC5727916

[B2] Altschuler E. L. Hud N. V. Mazrimas J. A. Rupp B. (1997). Random coil conformation for extended polyglutamine stretches in aqueous soluble monomeric peptides. J. Pept. Res. 50, 73–75. 10.1111/j.1399-3011.1997.tb00622.x 9273890

[B3] Babu M. M. (2016). The contribution of intrinsically disordered regions to protein function, cellular complexity and human disease. Biochem. Soc. Trans. 44, 1185–1200. 10.1042/bst20160172 27911701 PMC5095923

[B4] Babu M. M. Kriwacki R. W. Pappu R. V. (2012). Versatility from protein disorder. Science 337, 1460–1461. 10.1126/science.1228775 22997313

[B5] Ballatore C. Lee V. M. Trojanowski J. Q. (2007). Tau-mediated neurodegeneration in Alzheimer’s disease and related disorders. Nat. Rev. Neurosci. 8, 663–672. 10.1038/nrn2194 17684513

[B6] Banerjee P. R. Deniz A. A. (2014). Shedding light on protein folding landscapes by single-molecule fluorescence. Chem. Soc. Rev. 43, 1172–1188. 10.1039/c3cs60311c 24336839 PMC3958939

[B7] Banerjee P. R. Moosa M. M. Deniz A. A. (2016). Two-dimensional crowding uncovers a hidden conformation of α-synuclein. Angew. Chem. Int. Ed Engl. 55, 12789–12792. 10.1002/anie.201606963 27612332 PMC5166577

[B8] Bates G. Tabrizi S. Jones L. (2014). Huntington’s Disease. Oxford: Oxford University Press.

[B9] Bhattacharyya A. Thakur A. K. Chellgren V. M. Thiagarajan G. Williams A. D. Chellgren B. W.. (2006). Oligoproline effects on polyglutamine conformation and aggregation. J. Mol. Biol. 355, 524–535. 10.1016/j.jmb.2005.10.053 16321399

[B10] Birol M. Wojcik S. P. Miranker A. D. Rhoades E. (2019). Identification of N-linked glycans as specific mediators of neuronal uptake of acetylated α-synuclein. PLoS Biol. 17:e3000318. 10.1371/journal.pbio.3000318 31211781 PMC6599126

[B11] Borbat P. Ramlall T. F. Freed J. H. Eliezer D. (2006). Inter-helix distances in lysophospholipid micelle-bound α-synuclein from pulsed ESR measurements. J. Am. Chem. Soc. 128, 10004–10005. 10.1021/ja063122l 16881616

[B12] Brandt R. Leger J. Lee G. (1995). Interaction of tau with the neural plasma membrane mediated by tau’s amino-terminal projection domain. J. Cell Biol. 131, 1327–1340. 10.1083/jcb.131.5.1327 8522593 PMC2120645

[B13] Brucale M. Schuler B. Samori B. (2014). Single-molecule studies of intrinsically disordered proteins. Chem. Rev. 114, 3281–3317. 10.1021/cr400297g 24432838

[B14] Brunden K. R. Trojanowski J. Q. Lee V. M. (2009). Advances in tau-focused drug discovery for Alzheimer’s disease and related tauopathies. Nat. Rev. Drug Discov. 8, 783–793. 10.1038/nrd2959 19794442 PMC2787232

[B15] Burke K. A. Kauffman K. J. Umbaugh C. S. Frey S. L. Legleiter J. (2013). The interaction of polyglutamine peptides with lipid membranes is regulated by flanking sequences associated with huntingtin. J. Biol. Chem. 288, 14993–15005. 10.1074/jbc.m112.446237 23572526 PMC3663520

[B16] Burré J. Sharma M. Tsetsenis T. Buchman V. Etherton M. R. Südhof T. C. (2010). α-synuclein promotes SNARE-complex assembly *in vivo* and *in vitro*. Science 329, 1663–1667. 10.1126/science.1195227 20798282 PMC3235365

[B17] Bussell R. Jr. Eliezer D. (2003). A structural and functional role for 11-mer repeats in α-synuclein and other exchangeable lipid binding proteins. J. Mol. Biol. 329, 763–778. 10.1016/s0022-2836(03)00520-5 12787676

[B18] Butner K. A. Kirschner M. W. (1991). Tau protein binds to microtubules through a flexible array of distributed weak sites. J. Cell Biol. 115, 717–730. 10.1083/jcb.115.3.717 1918161 PMC2289193

[B19] Cabin D. E. Shimazu K. Murphy D. Cole N. B. Gottschalk W. McIlwain K. L.. (2002). Synaptic vesicle depletion correlates with attenuated synaptic responses to prolonged repetitive stimulation in mice lacking α-synuclein. J. Neurosci. 22, 8797–8807. 10.1523/jneurosci.22-20-08797.2002 12388586 PMC6757677

[B20] Chandra S. Chen X. Rizo J. Jahn R. Sudhof T. C. (2003). A broken α-helix in folded α-Synuclein. J. Biol. Chem. 278, 15313–15318. 10.1074/jbc.M213128200 12586824

[B21] Chattopadhyay K. Elson E. L. Frieden C. (2005). The kinetics of conformational fluctuations in an unfolded protein measured by fluorescence methods. Proc. Natl. Acad. Sci. U S A 102, 2385–2389. 10.1073/pnas.0500127102 15701687 PMC549012

[B24] Chen S. Berthelier V. Yang W. Wetzel R. (2001). Polyglutamine aggregation behavior *in vitro* supports a recruitment mechanism of cytotoxicity. J. Mol. Biol. 311, 173–182. 10.1006/jmbi.2001.4850 11469866

[B23] Chen J. Kanai Y. Cowan N. J. Hirokawa N. (1992). Projection domains of MAP2 and tau determine spacings between microtubules in dendrites and axons. Nature 360, 674–677. 10.1038/360674a0 1465130

[B22] Chen H. Rhoades E. (2008). Fluorescence characterization of denatured proteins. Curr. Opin. Struct. Biol. 18, 516–524. 10.1016/j.sbi.2008.06.008 18675353 PMC2628948

[B25] Cleveland D. W. Hwo S. Y. Kirschner M. W. (1977). Physical and chemical properties of purified tau factor and the role of tau in microtubule assembly. J. Mol. Biol. 116, 227–247. 10.1016/0022-2836(77)90214-5 146092

[B26] Cooper A. A. Gitler A. D. Cashikar A. Haynes C. M. Hill K. J. Bhullar B.. (2006). α-synuclein blocks ER-Golgi traffic and Rab1 rescues neuron loss in Parkinson’s models. Science 313, 324–328. 10.1126/science.1129462 16794039 PMC1983366

[B27] Cremades N. Cohen S. I. Deas E. Abramov A. Y. Chen A. Y. Orte A.. (2012). Direct observation of the interconversion of normal and toxic forms of α-synuclein. Cell 149, 1048–1059. 10.1016/j.cell.2012.03.037 22632969 PMC3383996

[B28] Crick S. L. Jayaraman M. Frieden C. Wetzel R. Pappu R. V. (2006). Fluorescence correlation spectroscopy shows that monomeric polyglutamine molecules form collapsed structures in aqueous solutions. Proc. Natl. Acad. Sci. U S A 103, 16764–16769. 10.1073/pnas.0608175103 17075061 PMC1629004

[B29] Crick S. L. Ruff K. M. Garai K. Frieden C. Pappu R. V. (2013). Unmasking the roles of N- and C-terminal flanking sequences from exon 1 of huntingtin as modulators of polyglutamine aggregation. Proc. Natl. Acad. Sci. U S A 110, 20075–20080. 10.1073/pnas.1320626110 24282292 PMC3864320

[B30] Crowther T. Goedert M. Wischik C. M. (1989). The repeat region of microtubule-associated protein tau forms part of the core of the paired helical filament of Alzheimer’s disease. Ann. Med. 21, 127–132. 10.3109/07853898909149199 2504257

[B31] Davidson W. S. Jonas A. Clayton D. F. George J. M. (1998). Stabilization of α-synuclein secondary structure upon binding to synthetic membranes. J. Biol. Chem. 273, 9443–9449. 10.1074/jbc.273.16.9443 9545270

[B32] Dedmon M. M. Lindorff-Larsen K. Christodoulou J. Vendruscolo M. Dobson C. M. (2005). Mapping long-range interactions in α-synuclein using spin-label NMR and ensemble molecular dynamics simulations. J. Am. Chem. Soc. 127, 476–477. 10.1021/ja044834j 15643843

[B33] Drescher M. Veldhuis G. van Rooijen B. D. Milikisyants S. Subramaniam V. Huber M. (2008). Antiparallel arrangement of the helices of vesicle-bound α-synuclein. J. Am. Chem. Soc. 130, 7796–7797. 10.1021/ja801594s 18512917

[B34] Drubin D. G. Kirschner M. W. (1986). Tau protein function in living cells. J. Cell Biol. 103, 2739–2746. 10.1083/jcb.103.6.2739 3098742 PMC2114585

[B35] Ebneth A. Godemann R. Stamer K. Illenberger S. Trinczek B. Mandelkow E. (1998). Overexpression of tau protein inhibits kinesin-dependent trafficking of vesicles, mitochondria and endoplasmic reticulum: implications for Alzheimer’s disease. J. Cell Biol. 143, 777–794. 10.1083/jcb.143.3.777 9813097 PMC2148132

[B37] Elbaum-Garfinkle S. Cobb G. Compton J. T. Li X. H. Rhoades E. (2014). Tau mutants bind tubulin heterodimers with enhanced affinity. Proc. Natl. Acad. Sci. U S A 111, 6311–6316. 10.1073/pnas.1315983111 24733915 PMC4035965

[B36] Elbaum-Garfinkle S. Rhoades E. (2012). Identification of an aggregation-prone structure of tau. J. Am. Chem. Soc. 134, 16607–16613. 10.1021/ja305206m 22998648 PMC3477793

[B38] Eliezer D. Kutluay E. Bussell R. Jr. Browne G. (2001). Conformational properties of α-synuclein in its free and lipid-associated states. J. Mol. Biol. 307, 1061–1073. 10.1006/jmbi.2001.4538 11286556

[B39] Ferreon A. C. Deniz A. A. (2007). α-synuclein multistate folding thermodynamics: implications for protein misfolding and aggregation. Biochemistry 46, 4499–4509. 10.1021/bi602461y 17378587

[B40] Ferreon A. C. Gambin Y. Lemke E. A. Deniz A. A. (2009). Interplay of α-synuclein binding and conformational switching probed by single-molecule fluorescence. Proc. Natl. Acad. Sci. U S A 106, 5645–5650. 10.1073/pnas.0809232106 19293380 PMC2667048

[B41] Ferreon A. C. Moran C. R. Ferreon J. C. Deniz A. A. (2010). Alteration of the α-synuclein folding landscape by a mutation related to Parkinson’s disease. Angew. Chem. Int. Ed Engl. 49, 3469–3472. 10.1002/anie.201000378 20544898 PMC2972640

[B42] Fersht A. R. (2008). From the first protein structures to our current knowledge of protein folding: delights and scepticisms. Nat. Rev. Mol. Cell Biol. 9, 650–654. 10.1038/nrm2446 18578032

[B43] Forster T. (1949). Experimentelle und theoretische untersuchung des zwischen-molekularen ubergangs von elektronenanregungsenergie. Z. Naturforsch. A 4, 321–327.

[B900] Gigant B. Landrieu I. Fauquant C. Barbier P. Huvent I. Wieruszeski J. M.. (2014). Mechanism of Tau-promoted microtubule assembly as probed by NMR spectroscopy. J. Am. Chem. Soc. 136, 12615–12623. 10.1021/ja504864m 25162583

[B44] Goedert M. (2001). α-synuclein and neurodegenerative diseases. Nat. Rev. Neurosci. 2, 492–501. 10.1038/35081564 11433374

[B45] Goode B. L. Chau M. Denis P. E. Feinstein S. C. (2000). Structural and functional differences between 3-repeat and 4-repeat tau isoforms. J. Biol. Chem. 275, 38182–38189. 10.1074/jbc.m007489200 10984497

[B46] Guo Q. Bin H. Cheng J. Seefelder M. Engler T. Pfeifer G.. (2018). The cryo-electron microscopy structure of huntingtin. Nature 555, 117–120. 10.1038/nature25502 29466333 PMC5837020

[B47] Gustke N. Trinczek B. Biernat J. Mandelkow E. M. Mandelkow E. (1994). Domains of tau protein and interactions with microtubules. Biochemistry 33, 9511–9522. 10.1021/bi00198a017 8068626

[B48] Hernández-Vega A. Braun M. Scharrel L. Jahnel M. Wegmann S. Hyman B. T.. (2017). Local nucleation of microtubule bundles through tubulin concentration into a condensed tau phase. Cell Rep. 20, 2304–2312. 10.1016/j.celrep.2017.08.042 28877466 PMC5828996

[B49] Hess S. T. Huang S. Heikal A. A. Webb W. W. (2002). Biological and chemical applications of fluorescence correlation spectroscopy: a review. Biochemistry 41, 697–705. 10.1021/bi0118512 11790090

[B50] Hirokawa N. Funakoshi T. Sato-Harada R. Kanai Y. (1996). Selective stabilization of tau in axons and microtubule-associated protein 2C in cell bodies and dendrites contributes to polarized localization of cytoskeletal proteins in mature neurons. J. Cell Biol. 132, 667–679. 10.1083/jcb.132.4.667 8647897 PMC2199865

[B51] Iakoucheva L. M. Brown C. J. Lawson J. D. Obradović Z. Dunker A. K. (2002). Intrinsic disorder in cell-signaling and cancer-associated proteins. J. Mol. Biol. 323, 573–584. 10.1016/s0022-2836(02)00969-5 12381310

[B52] Jakes R. Spillantini M. G. Goedert M. (1994). Identification of two distinct synucleins from human brain. FEBS Lett. 345, 27–32. 10.1016/0014-5793(94)00395-5 8194594

[B53] Jao C. C. Der-Sarkissian A. Chen J. Langen R. (2004). Structure of membrane-bound α-synuclein studied by site-directed spin labeling. Proc. Natl. Acad. Sci. U S A 101, 8331–8336. 10.1073/pnas.0400553101 15155902 PMC420394

[B54] Jeganathan S. von Bergen M. Brutlach H. Steinhoff H. J. Mandelkow E. (2006). Global hairpin folding of tau in solution. Biochemistry 45, 2283–2293. 10.1021/bi0521543 16475817

[B55] Johnson G. V. Stoothoff W. H. (2004). Tau phosphorylation in neuronal cell function and dysfunction. J. Cell Sci. 117, 5721–5729. 10.1242/jcs.01558 15537830

[B56] Joo C. Balci H. Ishitsuka Y. Buranachai C. Ha T. (2008). Advances in single-molecule fluorescence methods for molecular biology. Annu. Rev. Biochem. 77, 51–76. 10.1146/annurev.biochem.77.070606.101543 18412538

[B57] Klein F. A. C. Pastore A. Masino L. Zeder-Lutz G. Nierengarten H. Oulad-Abdelghani M.. (2007). Pathogenic and non-pathogenic polyglutamine tracts have similar structural properties: towards a length-dependent toxicity gradient. J. Mol. Biol. 371, 235–244. 10.1016/j.jmb.2007.05.028 17560603

[B58] König I. Zarrine-Afsar A. Aznauryan M. Soranno A. Wunderlich B. Dingfelder F.. (2015). Single-molecule spectroscopy of protein conformational dynamics in live eukaryotic cells. Nat. Methods 12, 773–779. 10.1038/nmeth.3475 26147918

[B59] Kriwacki R. W. Hengst L. Tennant L. Reed S. I. Wright P. E. (1996). Structural studies of p21Waf1/Cip1/Sdi1 in the free and Cdk2-bound state: conformational disorder mediates binding diversity. Proc. Natl. Acad. Sci. U S A 93, 11504–11509. 10.1073/pnas.93.21.11504 8876165 PMC38087

[B60] Kundel F. Tosatto L. Whiten D. R. Wirthensohn D. C. Horrocks M. H. Klenerman D. (2018). Shedding light on aberrant interactions—a review of modern tools for studying protein aggregates. FEBS J. 285, 3604–3630. 10.1111/febs.14409 29453901

[B61] Lee T. Moran-Gutierrez C. R. Deniz A. A. (2015). Probing protein disorder and complexity at single-molecule resolution. Semin. Cell Dev. Biol. 37, 26–34. 10.1016/j.semcdb.2014.09.027 25305580 PMC4339442

[B62] Lemke E. A. (2011). Site-specific labeling of proteins for single-molecule FRET measurements using genetically encoded ketone functionalities. Methods Mol. Biol. 751, 3–15. 10.1007/978-1-61779-151-2_1 21674321

[B64] Li X. H. Culver J. A. Rhoades E. (2015). Tau binds to multiple tubulin dimers with helical structure. J. Am. Chem. Soc. 137, 9218–9221. 10.1021/jacs.5b04561 26165802 PMC4697934

[B63] Li X. H. Rhoades E. (2017). Heterogeneous tau-tubulin complexes accelerate microtubule polymerization. Biophys. J. 112, 2567–2574. 10.1016/j.bpj.2017.05.006 28636913 PMC5479049

[B65] Litman P. Barg J. Rindzoonski L. Ginzburg I. (1993). Subcellular localization of tau mRNA in differentiating neuronal cell culture: implications for neuronal polarity. Neuron 10, 627–638. 10.1016/0896-6273(93)90165-n 8476613

[B66] Lv Z. Krasnoslobodtsev A. V. Zhang Y. Ysselstein D. Rochet J. C. Blanchard S. C.. (2015). Direct detection of α-synuclein dimerization dynamics: single-molecule fluorescence analysis. Biophys. J. 108, 2038–2047. 10.1016/j.bpj.2015.03.010 25902443 PMC4407253

[B67] MacDonald M. E. Ambrose C. M. Duyao M. P. Myers R. H. Lin C. Srinidhi L.. (1993). A novel gene containing a trinucleotide repeat that is expanded and unstable on Huntington’s disease chromosomes. Cell 72, 971–983. 10.1016/0092-8674(93)90585-e 8458085

[B68] Mangiarini L. Sathasivam K. Seller M. Cozens B. Harper A. Hetherington C.. (1996). Exon 1 of the HD gene with an expanded CAG repeat is sufficient to cause a progressive neurological phenotype in transgenic mice. Cell 87, 493–506. 10.1016/s0092-8674(00)81369-0 8898202

[B69] Maroteaux L. Campanelli J. T. Scheller R. H. (1988). Synuclein: a neuron-specific protein localized to the nucleus and presynaptic nerve terminal. J. Neurosci. 8, 2804–2815. 10.1523/jneurosci.08-08-02804.1988 3411354 PMC6569395

[B70] Melo A. M. Coraor J. Alpha-Cobb G. Elbaum-Garfinkle S. Nath A. Rhoades E. (2016). A functional role for intrinsic disorder in the tau-tubulin complex. Proc. Natl. Acad. Sci. U S A 113, 14336–14341. 10.1073/pnas.1610137113 27911791 PMC5167143

[B71] Melo A. M. Elbaum-Garfinkle S. Rhoades E. (2017). Insights into tau function and dysfunction through single-molecule fluorescence. Methods Cell Biol. 141, 27–44. 10.1016/bs.mcb.2017.06.010 28882307 PMC6209093

[B72] Melo A. M. Prieto M. Coutinho A. (2011). The effect of variable liposome brightness on quantifying lipid-protein interactions using fluorescence correlation spectroscopy. Biochim. Biophys. Acta 1808, 2559–2568. 10.1016/j.bbamem.2011.06.001 21683682

[B73] Middleton E. R. Rhoades E. (2010). Effects of curvature and composition on α-synuclein binding to lipid vesicles. Biophys. J. 99, 2279–2288. 10.1016/j.bpj.2010.07.056 20923663 PMC3042580

[B74] Mittag T. Kay L. E. Forman-Kay J. D. (2010). Protein dynamics and conformational disorder in molecular recognition. J. Mol. Recognit. 23, 105–116. 10.1002/jmr.961 19585546

[B75] Moosa M. M. Ferreon A. C. Deniz A. A. (2015). Forced folding of a disordered protein accesses an alternative folding landscape. Chemphyschem 16, 90–94. 10.1002/cphc.201402661 25345588 PMC4286261

[B76] Mukrasch M. D. Bibow S. Korukottu J. Jeganathan S. Biernat J. Griesinger C.. (2009). Structural polymorphism of 441-residue tau at single residue resolution. PLoS Biol. 7:e34. 10.1371/journal.pbio.1000034 19226187 PMC2642882

[B77] Murphy D. D. Rueter S. M. Trojanowski J. Q. Lee V. M. (2000). Synucleins are developmentally expressed and α-synuclein regulates the size of the presynaptic vesicular pool in primary hippocampal neurons. J. Neurosci. 20, 3214–3220. 10.1523/jneurosci.20-09-03214.2000 10777786 PMC6773130

[B78] Nagai Y. Inui T. Popiel H. A. Fujikake N. Hasegawa K. Urade Y.. (2007). A toxic monomeric conformer of the polyglutamine protein. Nat. Struct. Mol. Biol. 14, 332–340. 10.1038/nsmb1215 17369839

[B79] Nemani V. M. Lu W. Berge V. Nakamura K. Onoa B. Lee M. K.. (2010). Increased expression of α-synuclein reduces neurotransmitter release by inhibiting synaptic vesicle reclustering after endocytosis. Neuron 65, 66–79. 10.1016/j.neuron.2009.12.023 20152114 PMC3119527

[B80] O’Brien E. P. Morrison G. Brooks B. R. Thirumalai D. (2009). How accurate are polymer models in the analysis of Förster resonance energy transfer experiments on proteins? J. Chem. Phys. 130:124903. 10.1063/1.3082151 19334885 PMC2736576

[B81] Polymeropoulos M. H. Lavedan C. Leroy E. Ide S. E. Dehejia A. Dutra A.. (1997). Mutation in the α-synuclein gene identified in families with Parkinson’s disease. Science 276, 2045–2047. 10.1126/science.276.5321.2045 9197268

[B82] Rhoades E. Ramlall T. F. Webb W. W. Eliezer D. (2006). Quantification of α-synuclein binding to lipid vesicles using fluorescence correlation spectroscopy. Biophys. J. 90, 4692–4700. 10.1529/biophysj.105.079251 16581836 PMC1471845

[B83] Sathasivam K. Neueder A. Gipson T. A. Landles C. Benjamin A. C. Bondulich M. K.. (2013). Aberrant splicing of HTT generates the pathogenic exon 1 protein in Huntington disease. Proc. Natl. Acad. Sci. U S A 110, 2366–2370. 10.1073/pnas.1221891110 23341618 PMC3568346

[B84] Saudou F. Humbert S. (2016). The biology of Huntingtin. Neuron 89, 910–926. 10.1016/j.neuron.2016.02.003 26938440

[B85] Schuler B. Eaton W. A. (2008). Protein folding studied by single-molecule FRET. Curr. Opin. Struct. Biol. 18, 16–26. 10.1016/j.sbi.2007.12.003 18221865 PMC2323684

[B86] Schuler B. Soranno A. Hofmann H. Nettels D. (2016). Single-molecule FRET spectroscopy and the polymer physics of unfolded and intrinsically disordered proteins. Annu. Rev. Biophys. 45, 207–231. 10.1146/annurev-biophys-062215-010915 27145874

[B87] Schulte J. Littleton J. T. (2011). The biological function of the Huntingtin protein and its relevance to Huntington’s disease pathology. Curr. Trends Neurol. 5, 65–78. 22180703 PMC3237673

[B88] Shammas S. L. Garcia G. A. Kumar S. Kjaergaard M. Horrocks M. H. Shivji N.. (2015). A mechanistic model of tau amyloid aggregation based on direct observation of oligomers. Nat. Commun. 6:7025. 10.1038/ncomms8025 25926130 PMC4421837

[B89] Shen K. Calamini B. Fauerbach J. A. Ma B. Shahmoradian S. H. Serrano Lachapel I. L.. (2016). Control of the structural landscape and neuronal proteotoxicity of mutant Huntingtin by domains flanking the polyQ tract. Elife 18:18065. 10.7554/eLife.18065 27751235 PMC5135392

[B90] Sherman E. Itkin A. Kuttner Y. Y. Rhoades E. Amir D. Haas E.. (2008). Using fluorescence correlation spectroscopy to study conformational changes in denatured proteins. Biophys. J. 94, 4819–4827. 10.1529/biophysj.107.120220 18326651 PMC2397356

[B91] Sillen A. Barbier P. Landrieu I. Lefebvre S. Wieruszeski J. M. Leroy A.. (2007). NMR investigation of the interaction between the neuronal protein tau and the microtubules. Biochemistry 46, 3055–3064. 10.1021/bi061920i 17311412

[B92] Snead D. Eliezer D. (2014). α-synuclein function and dysfunction on cellular membranes. Exp. Neurobiol. 23, 292–313. 10.5607/en.2014.23.4.292 25548530 PMC4276801

[B93] Snead D. Eliezer D. (2019). Intrinsically disordered proteins in synaptic vesicle trafficking and release. J. Biol. Chem. 294, 3325–3342. 10.1074/jbc.rev118.006493 30700558 PMC6416451

[B94] Soranno A. Buchli B. Nettels D. Cheng R. R. Müller-Späth S. Pfeil S. H.. (2012). Quantifying internal friction in unfolded and intrinsically disordered proteins with single-molecule spectroscopy. Proc. Natl. Acad. Sci. U S A 109, 17800–17806. 10.1073/pnas.1117368109 22492978 PMC3497802

[B95] Spillantini M. G. Crowther R. A. Jakes R. Hasegawa M. Goedert M. (1998). α-Synuclein in filamentous inclusions of Lewy bodies from Parkinson’s disease and dementia with lewy bodies. Proc. Natl. Acad. Sci. U S A 95, 6469–6473. 10.1073/pnas.95.11.6469 9600990 PMC27806

[B96] Takahashi T. Kikuchi S. Katada S. Nagai Y. Nishizawa M. Onodera O. (2008). Soluble polyglutamine oligomers formed prior to inclusion body formation are cytotoxic. Hum. Mol. Genet. 17, 345–356. 10.1093/hmg/ddm311 17947294

[B97] Terwel D. Dewachter I. Van Leuven F. (2002). Axonal transport, tau protein, and neurodegeneration in Alzheimer’s disease. Neuromolecular Med. 2, 151–165. 10.1385/nmm:2:2:151 12428809

[B98] Thakur A. K. Jayaraman M. Mishra R. Thakur M. Chellgren V. M. Byeon I.-J.. (2009). Polyglutamine disruption of the huntingtin exon1 N-terminus triggers a complex aggregation mechanism. Nat. Struct. Mol. Biol. 16, 380–389. 10.1038/nsmb.1570 19270701 PMC2706102

[B99] Theillet F. X. Binolfi A. Bekei B. Martorana A. Rose H. M. Stuiver M.. (2016). Structural disorder of monomeric α-synuclein persists in mammalian cells. Nature 530, 45–50. 10.1038/nature16531 26808899

[B100] Tompa P. (2012). Intrinsically disordered proteins: a 10-year recap. Trends Biochem. Sci. 37, 509–516. 10.1016/j.tibs.2012.08.004 22989858

[B101] Trexler A. J. Rhoades E. (2009). α-synuclein binds large unilamellar vesicles as an extended helix. Biochemistry 48, 2304–2306. 10.1021/bi900114z 19220042 PMC2837115

[B102] Trexler A. J. Rhoades E. (2010). Single molecule characterization of α-synuclein in aggregation-prone states. Biophys. J. 99, 3048–3055. 10.1016/j.bpj.2010.08.056 21044603 PMC2965999

[B103] Trinczek B. Biernat J. Baumann K. Mandelkow E. M. Mandelkow E. (1995). Domains of tau protein, differential phosphorylation and dynamic instability of microtubules. Mol. Biol. Cell 6, 1887–1902. 10.1091/mbc.6.12.1887 8590813 PMC366657

[B104] Trojanowski J. Q. Lee V. M. (2005). Pathological tau: a loss of normal function or a gain in toxicity? Nat. Neurosci. 8, 1136–1137. 10.1038/nn0905-1136 16127446

[B105] Tsafou K. Tiwari P. B. Forman-Kay J. D. Metallo S. J. Toretsky J. A. (2018). Targeting intrinsically disordered transcription factors: changing the paradigm. J. Mol. Biol. 430, 2321–2341. 10.1016/j.jmb.2018.04.008 29655986

[B106] Uversky V. N. (2014). The triple power of D^3^: protein intrinsic disorder in degenerative diseases. Front. Biosci. *(Landmark Ed.)* 19, 181–258. 10.2741/4204 24389181

[B107] Uversky V. N. (2015). Intrinsically disordered proteins and their (disordered) proteomes in neurodegenerative disorders. Front. Aging Neurosci. 7:18. 10.3389/fnagi.2015.00018 25784874 PMC4345837

[B108] Uversky V. N. Oldfield C. J. Dunker A. K. (2008). Intrinsically disordered proteins in human diseases: introducing the D2 concept. Annu. Rev. Biophys. 37, 215–246. 10.1146/annurev.biophys.37.032807.125924 18573080

[B109] van der Lee R. Buljan M. Lang B. Weatheritt R. J. Daughdrill G. W. Dunker A. K.. (2014). Classification of intrinsically disordered regions and proteins. Chem. Rev. 114, 6589–6631. 10.1021/cr400525m 24773235 PMC4095912

[B110] Veldhuis G. Segers-Nolten I. Ferlemann E. Subramaniam V. (2009). Single-molecule FRET reveals structural heterogeneity of SDS-bound α-synuclein. Chembiochem 10, 436–439. 10.1002/cbic.200800644 19107759

[B1120] Warner J. B. T. Ruff K. M. Tan P. S. Lemke E. A. Pappu R. V. Lashuel H. A. (2017). Monomeric huntingtin exon 1 has similar overall structural features for wild-type and pathological polyglutamine lengths. J. Am. Chem. Soc. 139, 14456–14469. 10.1021/jacs.7b06659 28937758 PMC5677759

[B112] Wegmann S. Eftekharzadeh B. Tepper K. Zoltowska K. M. Bennett R. E. Dujardin S.. (2018). Tau protein liquid-liquid phase separation can initiate tau aggregation. EMBO J. 37:e98049. 10.15252/embj.201798049 29472250 PMC5881631

[B113] Weingarten M. D. Lockwood A. H. Hwo S. Y. Kirschner M. W. (1975). A protein factor essential for microtubule assembly. Proc. Natl. Acad. Sci. U S A 72, 1858–1862. 10.1073/pnas.72.5.1858 1057175 PMC432646

[B114] Weinreb P. H. Zhen W. Poon A. W. Conway K. A. Lansbury P. T. Jr. (1996). NACP, a protein implicated in Alzheimer’s disease and learning, is natively unfolded. Biochemistry 35, 13709–13715. 10.1021/bi961799n 8901511

[B115] Wellington C. L. Ellerby L. M. Gutekunst C. A. Rogers D. Warby S. Graham R. K.. (2002). Caspase cleavage of mutant huntingtin precedes neurodegeneration in Huntington’s disease. J. Neurosci. 22, 7862–7872. 10.1523/jneurosci.22-18-07862.2002 12223539 PMC6758089

[B116] Wetzel R. (2012). Physical chemistry of polyglutamine: intriguing tales of a monotonous sequence. J. Mol. Biol. 421, 466–490. 10.1016/j.jmb.2012.01.030 22306404 PMC3362671

[B117] Wickramasinghe S. P. Lempart J. Merens H. E. Murphy J. Huettemann P. Jakob U.. (2019). Polyphosphate initiates tau aggregation through intra- and intermolecular scaffolding. Biophys. J. 117, 717–728. 10.1016/j.bpj.2019.07.028 31400913 PMC6712485

[B118] Winklhofer K. F. Tatzelt J. Haass C. (2008). The two faces of protein misfolding: gain- and loss-of-function in neurodegenerative diseases. EMBO J. 27, 336–349. 10.1038/sj.emboj.7601930 18216876 PMC2234348

[B119] Wright P. E. Dyson H. J. (1999). Intrinsically unstructured proteins: re-assessing the protein structure-function paradigm. J. Mol. Biol. 293, 321–331. 10.1006/jmbi.1999.3110 10550212

[B120] Wright P. E. Dyson H. J. (2015). Intrinsically disordered proteins in cellular signalling and regulation. Nat. Rev. Mol. Cell Biol. 16, 18–29. 10.1038/nrm3920 25531225 PMC4405151

[B121] Zhang X. Lin Y. Eschmann N. A. Zhou H. Rauch J. N. Hernandez I.. (2017). RNA stores tau reversibly in complex coacervates. PLoS Biol. 15:e2002183. 10.1371/journal.pbio.2002183 28683104 PMC5500003

[B122] Zijlstra N. Blum C. Segers-Nolten I. M. Claessens M. M. Subramaniam V. (2012). Molecular composition of sub-stoichiometrically labeled α-synuclein oligomers determined by single-molecule photobleaching. Angew. Chem. Int. Ed Engl. 51, 8821–8824. 10.1002/anie.201200813 22806998

